# Dexmedetomidine in combination with sufentanil for postoperative analgesia after partial laryngectomy

**DOI:** 10.1186/s12871-017-0363-x

**Published:** 2017-05-25

**Authors:** Minju Qin, Kaizheng Chen, Tingjie Liu, Xia Shen

**Affiliations:** Department of Anesthesiology, The Eye, Ear, Nose and Throat Hospital of Fudan University, Shanghai Medical College of Fudan University, 83 fenyang road, Shanghai, 200031 China

**Keywords:** Analgesia, Patient-controlled, Sufentanil, Dexmedetomidine, Otolaryngological

## Abstract

**Background:**

Dexmedetomidine as an adjunct with opioids has been confirmed to spare opioids usage and improve analgesia for postoperative pain treatment. Furthermore, dexmedetomidine can attenuate the airway reflex. The aim of this study is to assess the safety and efficacy of dexmedetomidine combined with sufentanil for postoperative analgesia after partial laryngectomy.

**Methods:**

A total of 60 adult male patients were recruited and randomly allocated to receive sufentanil 1.0 μg ml^−1^ (Group S) or sufentanil 1.0 μg ml^−1^ plus dexmedetomidine 4 μg ml^−1^ (Group SD) for postoperative analgesia. The IV patient controlled analgesia (PCA) device was programmed to deliver 1.5 ml per demand with a 10 min lockout interval and 1.5 ml per hour background infusion. Cumulative consumption of sufentanil and pain intensity during 24 hour (h) after surgery were recorded. Coughing episodes per day, sleep quality, hemodynamic and respiratory profiles were measured.

**Results:**

Compared with Group S, patients in Group SD required less sufentanil during the 0–24 h postoperative period (*p* < 0.0001) and reported significant lower pain intensity from the second postoperative hour to the end of the study (*P* < 0.0001). Daily coughing episodes, sleep disturbance was lower and patients’ satisfaction was higher in Group SD (*P* < 0.05). Decrease in heart rate and mean blood pressure from baseline at 1 h, 2 h, 3 h, 12 h, and 24 h after operation were significantly greater in Group SD (*P* = 0.00). The incidence of PCA related adverse events were comparable between the two groups.

**Conclusion:**

Dexmedetomidine/sufentanil combination for postoperatjve analgesia in partial laryngectomized patients resulted in significant sufentanil sparing, better analgesia, reduced frequency coughing episodes, and better sleep quality.

**Trial registration:**

Chinese Clinical Registry (ChiCTR): ChiCTR-TRC-14004618, date of registration: 08 May 2014.

## Background

Maximizing pain relief, minimizing analgesic-related adverse events, and improving patients’ satisfaction are ideal to patient recovery after surgery. Pharyngolaryngeal cancer surgery is associated with a high level of pain in the early postoperative period [1]. The IV route is the common way of providing postoperative analgesia for pharyngolaryngeal surgery. After laryngectomy, the upper and lower airway tracts are separated. Colder and dryer air directly enters into the trachea and causes troublesome respiratory problems (i.e. excessive sputum production and involuntary coughing). Sleep disturbance is prevalent in these patients [2, 3]. The respiratory problems and poor sleep quality leads to low patients’ satisfaction.

Dexmedetomidine, a potent and highly selective α 2-adrenoreceptor agonist, as an adjunct with IV PCA opioids has been confirmed to improve analgesia, reduce morphine related side effects, improve sleep quality, and provide better patients’ satisfaction. [4–6]. Dexmedetomidine can attenuate airway irritation during airway manipulation [7]. The aim of this study was to evaluate whether dexmedetomidine added to sufentanil could reduce sufentanil use, enhance analgesia, ameliorate coughing, and improve sleep quality after pharyngolaryngeal surgery.

## Methods

This study was registered with the China clinical research information service in conformation with the tenets of the Declaration of Helsinki(ChiCTR-TRC-14004618, approval date: 2014/05/08). After obtaining approval from the hospital ethics committee (Shanghai Eye & ENT hospital, Fudan university) and written informed consent from patients, we enrolled 60 adult patients with American Society of Anesthesiologist physical status I or II, aged 40–65 years, undergoing partial laryngectomy. Exclusion criteria were patients with hypertension, ischemia heart disease, taking β-adrenoreceptor blockers or analgesics or tranquilizers during the last weeks, history of postoperative nausea and vomiting, or a known allergic to any of the medication used perioperatively. Female patients were also excluded.

A computer-generated randomization table was used to allocate patients into two groups (*n* = 30 per group). The 150 ml solution in the PCA reservoir bag contained 150 μg of sufentanil in normal saline (1.0 μg ml^−1^) in Group S or 150 μg sufentanil plus 600 μg dexmedetomidine in normal saline (sufentanil 1.0 μg ml^−1^, dexmedetomidine 4 μg ml^−1^) in Group SD. Both patients and observers were blinded to the allocation. Double-blinding was achieved by labeling the PCA reservoir bags with a particular identification number only.

The day before surgery, all patients were instructed on the operational use of PCA device (apon Medical technology Co., Ltd, Jiangsu, China) and a standard visual analogue scale (VAS) for pain (0, no pain; 10, the worst pain intolerable). In the holding room on surgery day, the use of the PCA device and VAS was explained to the patients again. In the operating room, patients were connected to standard American Society of Anesthesiologists monitors. A peripheral venous cannula was inserted into the forearm for 0.9% normal saline infusion at a rate a 400 ml.h^−1^. Anesthesia induction began with propofol 2.5 mg kg^−1^, sufentanil 0.3 μg kg^−1^, and rocuronium 0.6 mg kg^−1^. After confirmation of successful tracheal intubation, patients received pressure controlled ventilation. Prior to the start of surgery, IV boluses of parecoxib 1.0 mg kg^−1^, sufentanil 0.1 μg kg^−1^, ondansetron hydrochloride 0.15 mg kg^−1^ was given. Anesthesia was maintained with sevoflurane 1 MAC in 40% oxygen/air mixture. Before laryngectomy, another sufentanil 0.1 μg kg^−1^ and a bolus of dexmedetomidine (0.5 μg kg^−1^, finished in 10 min by infusion) was administered. At the end of the surgery, neostigmine 0.04 mg kg^−1^ and atropine 0.02 mg kg^−1^ were given to reverse residual neuromuscular block. Steroids were not used in the operative period. When the patient recovered from anesthesia, the first evaluation regarding pain intensity was performed and the time was defined as hour 0 (H0).

Patients were transferred to the post-anesthesia care unit (PACU) for further recovery. On arrival at PACU, patients were connected to the IV PCA device. The setting for PCA was 1.5 ml bolus (containing sufentanil 1.5 μg or sufentanil/dexmedetomidine 1.5 μg/6 μg) and lockout time of 10 min with background continuous infusion 1.5 ml per hour. When patients fulfilled the criteria of the modified Aldrete score [8], they were transferred to the intensive care unit. Humidified and heated oxygen/air was delivered (Fio2: 40%) via the laryngeal cannula. If the patient reported a VAS of 5 or higher at rest, an anesthesiologist not involved in the study gave the patient 0.1 mg kg-1 morphine intravenously for pain rescue.

Baseline heart rate (HR) and mean blood pressure (MBP) were documented after ward admission. A nurse anesthetist blinded to the study recorded the following variables in all patients at 1 h, 2 h, 3 h, 12 h, and 24 h after operation; pain intensity at rest (VASR) and then after swallowing (VASS) and the hemodynamic and respiratory profiles. Total sufentanil consumption in the first 24 hour was recorded. Each patient was asked concerning the daily coughing episodes, sleep disturbance, and satisfaction with pain treatment. Patients were also questioned as to the presence of nausea, vomiting, pruritus and difficult voiding. Severe sedation was defined as a Ramsay Scale greater than 3. Hypotension was defined as more than 20% decrease in MBP from preoperative baseline. Bradycardia was defined as heart beat < 60 min^−1^. Respiratory depression was defined as ventilatory rates less than 8 min^−1^ or SpO_2_ < 90%. Hypotension or bradycardia was treated with volume expansion, ephedrine, or atropine. Respiratory depression was treated with naloxone and oxygen.

The power calculation for the study was based on sufentanil consumption in the first 24 h after surgery. Based on a pilot study, the average sufentanil requirement was 45 (SD 10) μg in 24 h. To detect an estimate of a 15% reduction in sufentanil use in the first 24 h after surgery, it was estimated that a sample size of 20 patients would be required to detect a significant difference at the 0.05 level with a power of 0.8. In this study we enrolled 60 patients to improve the power of test. Sufentanil consumption in the first 24 hour was the primary outcome parameter, and all other measured parameters were considered secondary outcome parameters.

Data are reported as mean ± SD, unless otherwise noted. Continuous data (age, BMI, sufentanil consumption) were analyzed with an unpaired Student’s *t*-test. Ordinal data (pain intensity) were analyzed using the Mann–Whitney ranked-sum test. Nominal data (coughing episodes, sleep quality, patient satisfaction, and adverse events) were analyzed using either *x*2 or Fisher’s exact test. Bonferroni-corrected post hoc test was conducted to adjust the observed significant level for multiple test. Interaction between time and group factors in a two-way analysis of variance (ANOVA) with repeated measurements was used to analyze hemodynamic profile (i.e. HR, MAP) between patients in Group S and in Group SD. The *post hoc* Bonferroni test was used to compare differences at different time points. A probability level of < 0.05 was considered to be statistically significant.

## Results

**Fig. 1:**
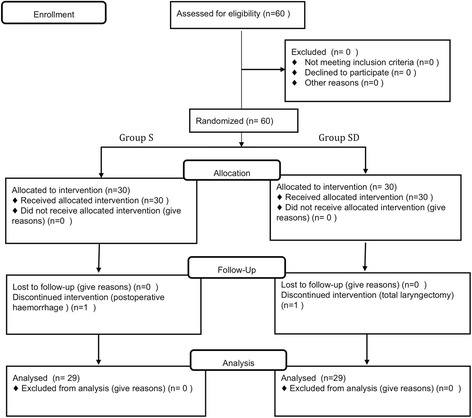
CONSORT flow diagram

A total of 60 adult male patients (between May 2014 and November 2014) were recruited in this study. All patients had a smoke history longer than 20 years. One patient in Group S required reoperation because of postoperative hemorrhage and the other one in Group SD needed total laryngectomy during the operation. Thus, 58 patients completed this study (Fig. 1).

There were no significant differences with regard to patient characteristics and intraoperative variables between two groups (Table 1). No patient needed morphine for pain rescue during the study. Neither did any patient need naloxone for rescue from sufentanl. In the first 24 hour, patients in Group SD required less sufentanil than those in Group S (38.0 μg vs 47.8 μg, *p* < 0.001). Pain intensities VASR and VASS were consistently lower in Group SD than in Group S from the second hour after operation (Table 2).

**Table 1:** Patients’ characteristics

Parameter	Group S (*n* = 29)	Group SD (*n* = 29)
Age (years)	58.2 ± 6.5	58.5 ± 6.2
Weight (kg)	68.2 ± 6.9	67.8 ± 8.5
BMI (kg/m^2^)	23.8 ± 1.8	23.2 ± 2.6
Duration of surgery (min)	104.3 ± 7.8	103.9 ± 9.2
Sufentanil dose (μg)	34.1 ± 3.5	33.9 ± 4.3
Blood loss (ml)	160.7 ± 20.3	164.8 ± 18.2
0.9% normal saline infusion (ml)	1367.2 ± 122.7	1375.9 ± 102.3

Values are mean (SD)

*S* sufentanil group, *SD* sufentanil/dexmedetomidine group, *BMI* body mass index

**Table 2:** Sufentanil consumption and pain intensity at rest and on swallow during the first 24 hour

Parameter	Group S (*n* = 29)	Group SD (*n* = 29)
Sufentanil consumption (μg)	47.8 ± 4.7	38.0 ± 1.8**
At rest		
0 h	2 (1–2)	2 (1–2)
1 h	2 (1–2)	1 (1–2)
2 h	2 (2–3)	1 (0.5–1)***
3 h	3 (2–3)	1(1–2)***
12 h	3 (2–4)	1 (1–2)***
24 h	2 (2–3)	1 (0.5–1)***
On swallow		
1 h	5 (4–5)	4 (3–5)
2 h	6 (5–7)	4 (3–5)***
3 h	6 (5–7)	4 (3.5–5)***
12 h	5 (4–5)	3 (3–4)***
24 h	4 (3–5)	3 (3–4)**

Values are mean (SD), median (inter-quartile range)

** *p* < 0.001, *** *p* < 0.0001, Group SD vs Group S

*S* sufentanil group, *SD* sufentanil/dexmedetomidine group, *VASR* visual analogue scale at rest, *VASS* visual analogue scale on swallow

In Group SD, twenty-two patients had one to five spontaneous coughing episodes per day, whereas seven patients had six to ten, and two patients had more than ten such episodes per day. In Group S, twelve patients had one to five spontaneous coughing episodes per day, ten patients had six to ten, and seven patients had more than ten such episodes per day, (*P* = 0.0453). Eighteen patients in Group SD, and nine patients in Group S did not complain sleeping disturbance during PAC treatment (*P* = 0.0343). Twenty-seven patients’ were satisfied with the PCA treatment and two liked it somewhat in Group SD. In Group S, nineteen patients were satisfied with the PCA, nine liked it somewhat, and one reported that PCA did not help them (*P* =0.0326) (Table 3).

**Table 3:** Coughing, sleep quality, and patients’ satisfaction

	Group S (*n* = 29)	Group SD (*n* = 29)
Coughing episode		
1–5	12 (41.4)	22 (75.9)
6–10	10 (34.5)	7 (24.1)
>10	7 (24.1)	2 (6.9)
Sleeping disturbance	20 (69.0)	11 (37.9)
Satisfaction		
Favorable	19 (65.5)	27 (93.1)
A little	9 (31.0)	2 (6.9)
Not favorable	1 (3.4)	0

Values are given as number of subjects (%)

*S* sufentanil group, *SD* sufentanil/dexmedetomidine group

**Fig. 2:**
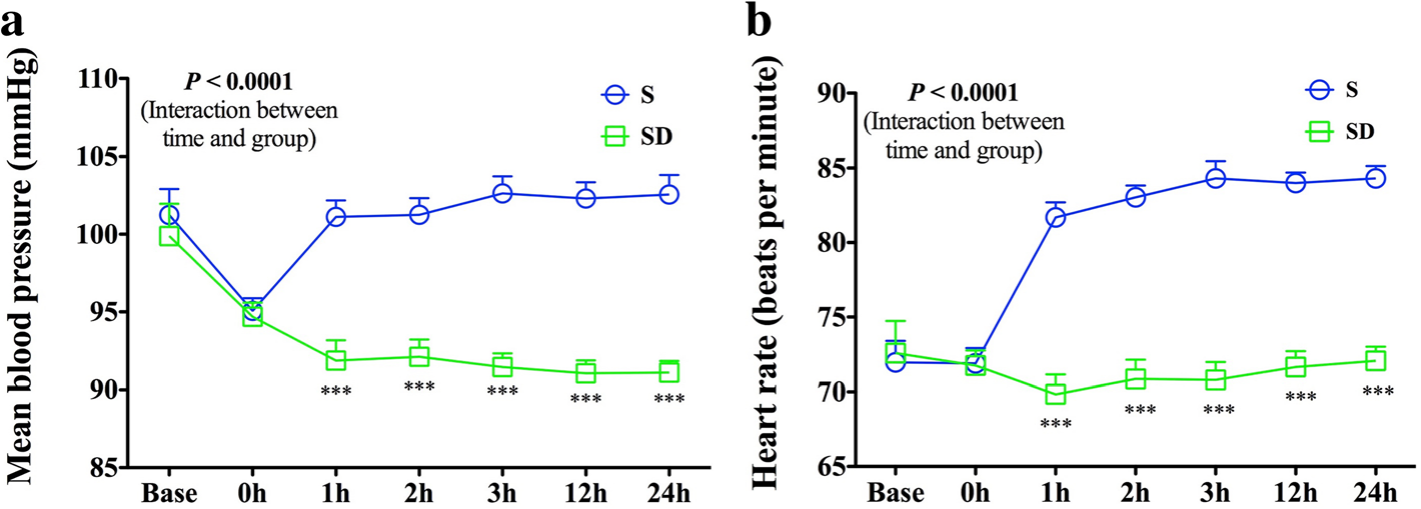
Changes in MBP (**a**) and HR (**b**) after the surgery. Repeated-measurement ANOVA with post hoc Bonferroni test showed significant decrease in MAP (**a** *P* < 0.0001) over times between Group S and Group SD. There was also a significantly decreased HR (**b** *P* < 0.0001) in the SD group vs S groups over time. ***P < 0.0001

Regarding hemodynamic variables, patients in Group SD had lower MAP and slower HR than in Group S after operation. Repeated-measurement ANOVA with *post hoc* Bonferroni test showed a difference in MAP over times between the two groups, with MAP at 1 h, 2 h, 3 h, 12 h, and 24 h being significantly lower in Group SD than in Group S (Fig. 2a, *P* < 0.0001). HR was significantly slower over times (Fig. 2b, *P* < 0.0001, two-way ANOVA with repeated measurement). The *post hoc* Bonferroni test showed that patients in Group SD had slower HR at 1 h, 2 h, 3 h, 12 h, and 24 h being significantly slower in Group SD than in Group S. No patient experienced hypotension and bradycardia.

Main adverse events were reported in Table 4. The overall (0–24 h) incidence of nausea and vomiting was not significantly between two groups SD (20.6% vs 17.2%, *p* = 1). Patients in two groups reported similar pruritus (20.6% vs 10.3%, *p* = 0.47). There was no report of respiratory depression in this study. No severe sedation was observed in the two groups.

**Table 4:** Incidence of adverse events

Parameter	Group S (*n* = 29)	Group SD (*n* = 29)
Nausea	6 (20.6)	5 (17.2)
Vomiting	2 (6.8)	3 (10.3)
Pruritus	6 (20.6)	3 (10.3)
Uninary retention	0	0
Severe sedation	0	0

Values are given as number of subjects (%)

*S* sufentanil group, *SD* sufentanil/dexmedetomidine group

## Discussion

The study showed that dexmedetomidine-sufentanil mixture significantly reduced sufentanil requirement, enhanced the analgesic effect of sufentanil, reduced coughing episodes and improved sleep quality, without clinically relevant respiratory depression or over-sedation.

Our finding that patients receiving dexmedetomidine required 20.6% less PCA sufentanil is in line with studies showing opioids-sparing effects by dexmedetomidine [4, 9, 10]. Lin et al. [4] pointed out that improved analgesia by dexmedetomidine might come from the synergic analgesic interaction with opioids, reduction of stress, and attenuation on the affective-motivational component of pain. In our study, either in S group or SD group, the patients’ VASRs were less than 4 at different time points in both groups. But patients receiving dexmedetomidine-sufentanil mixture had lower pain scores on swallow than those receiving sufentanil alone. We thought this difference was of clinical significance.

The patients undergoing partial laryngectomy suffer profound physiological changes due to the separation of upper and lower airway tract. The patients all had a long history of smoke for more than 20 years. They were at risk of developing chronic bronchitis and airway hyper-reaction. Airway irritation is more likely when unconditioned air enters into the trachea and bronchi. Troublesome respiratory problems (i.e. excessive sputum production and incessant coughing) usually result in poor sleep [2]. Dexmedetomidine is widely used in the mechanically ventilated patients in ICU for sedation and better sleep quality [6]. In our study, we found that the patients with dexmedetomide had less sleep disturbance. Furthermore, fewer patients in group SD have frequent coughing episodes. The reasons might be: First, opioids and dexmedtomidine both possess the characteristics of attenuating airway reflex [11, 12], when they are combined, the effect is synergic. Second, dexmedetomidine might improve the compliance with the heat and humidification device, so that airway was devoid of irritation from the cold and dry air.

As for hemodynamic profiles, dexmedetomidine-sufentanil mixture caused greater decrease in HR and MAP from pre-surgery baseline at 1 h, 2 h, 3 h, 12 h, and 24 h after operation in SD group. The magnitude of decrease in HR and MAP was not clinically significant as it did not lead to bradycardia or hypotension. Good sleep quality and the sympatho-inhibitory effect of dexmedetomidine and sufentanil both benefit hemodynamic stability [6].

Sufentanil combination with dexmedetomidine for PCA after surgery might produce untoward sedation. In our study, we did not found over-sedation in patients receiving dexmedetomidine-sufentanil mixture. The reasons were as follow: First, the dose of dexmedetomidine infusion throughout the study was less than 0.1 μg kg h^−1^, this dose was below the range of the recommended 0.2–0.7 μg kg h^−1^, maintenance infusion for intensive care sedation. Second, the reduced cumulative PCA sufentanil requirements could also help mitigate sedation.

There are limitations in our study. First, we subjected the patients to a continuous infusion of sufentanil or sufentanil/dexmedetomidine – combined with PCA demand boluses. This is not in essence a true PCA-regimen. Considering the pharmacologic effect of opioids and dexmedetomidine for attenuating airway reflex, we applied continuous infusion. Second, we did not evaluate the extent of anxiety. It is known that psychological issues are common in laryngectomized patients [13]. Third, we planned to measured pain intensity at swallow in our study. In our institution, patients undergoing total laryngectomy are not encouraged to swallow just in case pharyngo-cutancous fistula occurs. We did not enroll the patients undergoing complete laryngectomy. Fourth, we did not observe the gag ability which may be blunted by opioids. The laryngectomized patients routinely had nasogastric feed tube. Even if swallow function was impaired by surgery and opioids, the patients could be feed on via the gastric tube. Besides, with the tracheostomy tube, there was no risk of airway aspiration. Fifth, we did not observe the effect of smoking on postoperative coughing. Despite the limitations metioned above, nowadays, anesthesiologists in our institution prefer sufentanil/dexmedetomidine for PCA in patients undergoing total or partial laryngectomy.

## Conclusion

In summary, our study demonstrated that IV PCA dexmedetomidine and sufentanil mixture reduced sufentanil requirement, enhanced analgesia, attenuated coughing, and improved sleep quality and patients satisfaction compared to PCA sufentanil alone.

## Abbreviations

ASA: American society of anesthesiologist
PCA: Patient controlled analgesia
VAS: Visual analogue scale
PACU: Post-anesthesia care unit
MAP: Mean blood pressure
HR: Heat beat
